# Supplementary material for: Association between Meteorological Factors and Mumps and Models for Prediction in Chongqing, China
Source: Int J Environ Res Public Health. 2022 May 29;19(11):6625. doi: 10.3390/ijerph19116625 (PMC9180516; doi:10.3390/ijerph19116625)
Supplement: Supplementary file 1 [file ijerph-19-06625-s001.zip › ijerph-1717729-supplementary.pdf]

Table S1. Overall demographic characteristics of mumps cases from 2009-2019 in Chongqing.

variables	Case number	Proportion (%)
Gender		
male	74089	58.29
female	53017	41.71
total	127107	100
Age		
0~	115358	90.76
18~	6751	5.31
35~	4092	3.22
60~	906	0.71
total	127107	100
Group		
workers	9986	7.86
housework and unemployment	482	0.38
preschool children	40229	31.65
students	75943	59.75
others	467	0.37
total	127107	100
